# The Rationale for Using Corticosteroids in COVID-19 Encephalopathy: Lessons From a Case Report With Evidence From Literature

**DOI:** 10.7759/cureus.33233

**Published:** 2023-01-01

**Authors:** Muhammad Atif Ameer, Haroon Chaudhry, Maham Babar, Nimi Patel, Monata Song, Mathew Mathew

**Affiliations:** 1 Department of Medicine, Punjab Rangers Teaching Hospital, Lahore, PAK; 2 Internal Medicine, Suburban Community Hospital, East Norriton, USA; 3 Department of Medicine, Khyber Medical University, Peshawar, PAK; 4 Internal Medicine, Krupa Hospital, Ahmedabad, IND; 5 Internal Medicine, Philadelphia College of Osteopathic Medicine, Philadelphia, USA; 6 Internal Medicine, Suburban Community Hospital, Philadelphia, USA; 7 Internal Medicine, Suburban Hospital, Norristown, USA

**Keywords:** neurological manifestation of covid, hospitalization in covid-19, covid-19 cns involvement, covid treatment, covid-19 pandemic, corticosteroids in covid-19, covid-19 management, remdesivir, covid-19-related encephalopathy

## Abstract

The coronavirus disease 2019 (COVID-19) virus primarily affects the pulmonary system, but neurological manifestations and complication of COVID-19 has been reported in abundance in the literature. We present a case of a middle-aged Caucasian male who was brought to the emergency department for altered mental status. His chief complaints were neurological rather than respiratory. A positive severe acute respiratory syndrome coronavirus 2 (SARS-CoV-2) reverse transcription polymerase chain reaction (RT-PCR) nasal swab confirmed the diagnosis. Brain imaging showed mildly dilated ventricles with no other acute findings. As the patient did not require oxygen, he was treated with remdesivir alone without corticosteroids, which is also a precipitating factor of psychosis but, unfortunately, thickly used in practice. That led to remarkable results in full recovery without exposing the patient to steroid therapy. We strongly believe that remdesivir alone is sufficient in treating COVID-19-induced encephalopathy in a patient who does not require oxygen, and evidence supports this practice.

## Introduction

Coronavirus disease 2019 (COVID-19) is caused by the highly contagious severe respiratory distress syndrome coronavirus 2 (SARS-CoV-2) and is prevalent in all age groups. COVID-19 was announced as a global health pandemic on 11 March 2020 after it emerged from the province of Wuhan, China, in December 2019. It has infected more than 654 million people, with a death toll greater than 6.65 million to date [1]. The novel coronavirus primarily affected the respiratory and cardiovascular systems. However, frequent neurological manifestations and complications of COVID-19 infection have been reported in the literature, including headache, anosmia/hyposmia, acute myelitis, encephalopathy, encephalitis, acute hemorrhagic necrotizing encephalopathy, and cerebrovascular accidents. SARS-CoV2 primarily attacks the respiratory epithelium. It invades the human host cells by binding to the cellular receptor angiotensin-converting enzyme 2 (ACE2) and by serine proteases TMPRSS2 for spike (S) protein binding. Although SARS-CoV-2 was not considered neurotropic in the early phase, invasion of the ACE2 receptors in glial cells and spinal neurons is postulated to be one of the reasons for the neurological manifestation of the COVID-19 infection. Animal models predicted the spread of SARS-CoV-2 through the olfactory bulb, reaching the central nervous system (CNS) [2]. We report a case of COVID-19 encephalopathy treated with remdesivir alone, aligning the guidelines without the unnecessary use of corticosteroids. Unfortunately, steroids are massively used in inpatient settings for COVID-positive patients who do not require oxygen. This practice usually leads to undesirable complications and is not supported by the evidence.

## Case presentation

A 50-year-old Caucasian male with a past medical history of schizophrenia and Type 2 diabetes mellitus was brought to the emergency department (ED) with altered mental status. The patient works in a supervised facility with the mentally challenged and had complained to his colleagues about cough and nasal congestion that started a few days ago. His coworker had not been able to reach him for two days. The patient did not show up for work on the day of the presentation. The coworker went to check up on him at his home and found him in a state of confusion and paranoia, and he was brought to ED. His home medications include metformin 500mg BID (twice a day), gabapentin 300 mg QD (four times a day), buspirone 30 mg BID, and clozapine 50 mg in the morning and afternoon and 600 mg at night (used for resistant schizophrenia). The patient had three prior episodes of clozapine toxicity, where he presented with agitation, hyperactivity, confusion, and diaphoresis.

On presentation, his blood pressure was 124/89 mmHg, heart rate 95/min, respiratory rate 18/min, and saturating 98% on room air. On evaluation, the patient was calm, disheveled, and unable to provide adequate history. Upon neurological examination, the patient was only oriented to himself. Pupils are equally reactive to light and accommodation. Extraocular motions were intact. The neck was soft, supple, and had a full range of motion without meningismus. Cranial nerves were grossly II-XI intact. Deep tendon reflexes were 2+. Bulk, muscle tone, and sensations were preserved throughout. Strength was assessed to be 4/5 in all four extremities. Mini-mental status score was predicted to be 18/30. On psychiatry evaluation, the patient was guarded with poor judgment and attention span, mild depression, impaired memory with a paranoid thought process. Multi-axial system analysis revealed schizophrenia and tobacco use disorder.

Initial laboratory analysis included a normal complete blood count (Table 1) and a comprehensive metabolic profile significant for acute kidney injury (AKI) (Table 2).

**Table 1:** Complete blood count (CBC)

Complete Blood Count			
White Blood Cells (WBC)	8.6 (Normal 4.5-11.0 × 10^9/L)	Mean corpuscular volume (MCV)	83.1 (Normal 80–100 fl)
Red Blood Cells (RBC)	5.28 (Normal 4.7- 6.1 cells/mcL)	Mean corpuscular hemoglobin (MCH)	28.2 (Normal 27.5-33.2 pg)
Hemoglobin (Hgb)	14.9 (Normal 13.8-17.2 g/dL)	Mean corpuscular hemoglobin concentration (MCHC)	33.9 (Normal 32 to 36 g/dL)
Hematocrit (Hct)	43.9 (Normal 41%-50%)	Red blood cell distribution width (RDW)	15.2 (Normal 11.8-14.5 %)
Platelets (Plts)	216 (Normal 150-400 × 10^9/L)	Mean platelet volume (MPV)	7.7 (Normal 7.2-11.7 fL)

**Table 2:** Comprehensive metabolic panel (CMP)

Comprehensive Metabolic Panel			
Sodium	137 (Normal 135 -145 mEq/L)	Protein	6.7 (Normal 6.0-8.3 g/dL)
Potassium	4.7 (Normal 3.5-5.0 mEq/L)	Albumin	6.7 (Normal 3.4 to 5.4 g/dL)
Chloride	103 (Normal 96-106 mEq/L)	Bilirubin	0.6 (Normal 0.1-1.2 mg/dL)
Bicarbonate	25.7 (Normal 23-29 mEq/L)	Globulin	2.8 (Normal 2.0-3.5 g/dL)
Anion Gap	8.3 (Normal 4-12 mmol/L)	Phosphorus	4.5 (Normal 2.8-4.5 mg/dL)
Blood Urea Nitrogen (BUN)	13 (Normal 6-24 mg/dL)	Magnesium	1.9 (Normal 1.3-2.1 mEq/L)
Creatinine (Cr)	1.9 (Normal 0.7 - 1.3 mg/dL)	Calcium	9.4 (Normal 8.5-10.2 mg/dL)
Glucose	240 (Normal 70-100 mg/dL)		
Aspartate transaminase (AST)	20 (Normal 8-33 U/L)		
Alanine transaminase (ALT)	21 (Normal 7-56 U/L)		
Alkaline Phosphatase (Alk Phos)	75 (Normal 30-120 IU/L)		

**Figure 1:**
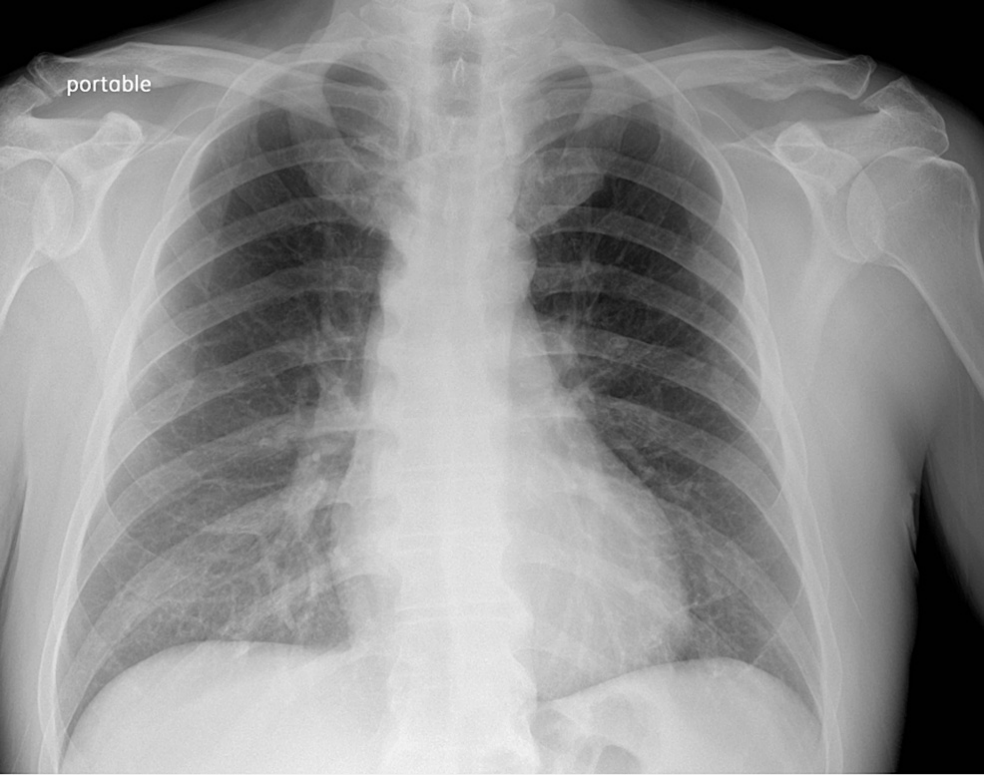
Chest X-ray The chest X-ray image shows no infiltrates or masses. No abnormality of hilar and mediastinal structures is appreciated.

**Figure 2:**
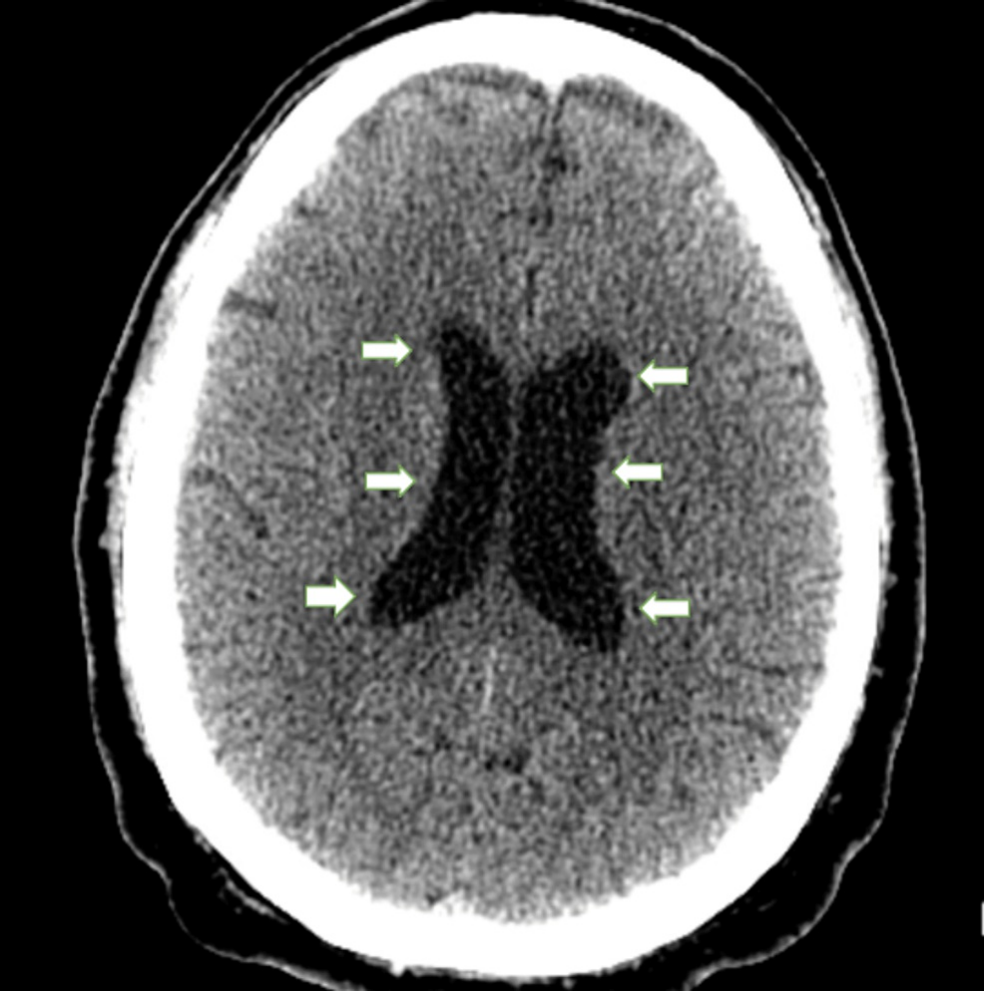
Head CT without contrast White arrows pointing towards dilated ventricles. No evidence of intracranial hemorrhage, midline shift, mass, infarcts, or acute changes were appreciated.

Chest X-ray (Figure 1) revealed no acute cardio-pulmonary abnormality. CT scan of the head without contrast (Figure 2) showed "No evidence of intracranial hemorrhage, and ventricles were slightly prominent, with no extra-axial fluid collections, or midline shifts noted. No hemorrhage, mass, or infarct."

Urinary drug screen was negative for a tricyclic antidepressant (TCA), benzodiazepines, barbiturates, opiates, cannabis, methadone, phencyclidine (PCP), oxycodone, propoxyphene, and amphetamine. The patient screened positive for COVID-19 via nasal swab. Clozapine levels were pending at this time. A provisional diagnosis of clozapine toxicity and COVID-19 encephalopathy was made, and the patient was initiated on supportive therapy. Over the next 24 hours, the patient's mentation remained unchanged.

The patient's COVID-19 PCR result came positive, and clozapine levels were within an acceptable range on the second day of admission. Clozapine toxicity was ruled out, and the patient was restarted on his home dose of clozapine. However, there was no improvement in the patient's mentation, and it was decided to start intravenous remdesivir 200 mg for one day as a loading dose and 100 mg for the next four days with a total course of five days according to the guidelines. Systemic steroids were deferred as per National Institutes of Health (NIH) guidelines as the patient maintained oxygen saturations of >97% on room air.

Within 24 hours of starting remdesivir, we observed improvement in mentation. The patient continued to show marked improvement in his mentation over the successive days of treatment. On the fifth day of treatment, the patient was found to have reasonable judgment, restored memory and concentration, and intact association with the improved thought process and cognitive function, eventually returning to his baseline. The patient completed a five-day course of remdesivir and was discharged shortly thereafter. He was referred to follow up with an outpatient psychiatry clinic.

## Discussion

SARS-CoV-2 is a highly contagious virus. Coronaviruses (CoVs) belong to the order Nidovirales, the family Coronaviridae, and the subfamily Coronavirinae. CoVs are a highly diverse family of enveloped positive-sense single-stranded RNA viruses. Amongst the structural proteins of SARS-CoV-2, the S-spike glycoprotein is the most integral. This protein is inactive until it is cleaved by peptidases into S1, which mediates adherence to host receptor molecules, and S2, which promotes the fusion of SARS-CoV-2 with host cell membranes. The S-spike protein interacts with many host cell receptor molecules, some of the most studied being ACE2, CD147, and GRP78 [2-3].

Angiotensin-converting enzyme 2 (ACE2) is a transmembrane protein composed of an extracellular domain which is the SARS-CoV-2 binding site. There are two forms of ACE2 protein, a cellular form and a circulating form. Cellular ACE2 protein is a membrane-bound full-length protein expressed abundantly in pneumocytes, enterocytes of the small intestine, and vascular endothelial cells of the heart, kidneys, and brain. Circulating ACE2 protein is cleaved from the cellular form by the metalloprotease ADAM17 and then released into the extracellular environment. TMPRSS2, a transmembrane serine protease, competes with ADAM17 for ACE2 cleavage. TMPRSS2-mediated cleavage leads to the release of cellular ACE2, which results in SARS-CoV-2-cell membrane fusion with augmented SARS-CoV-2 entry into the host cell. SARS-CoV-2 RNA is then released into the cytoplasm, and viral replication ensues. Studies suggest that certain brain areas, like the frontal operculum, para-olfactory gyrus, and olfactory tubercles, demonstrate overexpression of TMPRSS2 [2-4].

The main target of SARS-CoV-2 is the respiratory system. However, a wide distribution of ACE2 receptors leads to cardiovascular, gastrointestinal, and central nervous system involvement. Although the more frequently reported neurological manifestations of SARS-CoV-2 are headache, dizziness, anosmia, and ageusia, severe neurological complications like encephalopathy have also been reported [5]. According to one meta-analysis that evaluated neurological outcomes in 1643 SARS-CoV-2 patients, the overall incidence of encephalopathy was 9.14% (95% CI:2.20 to 19.81) [6]. The hallmark of encephalopathy is diffuse brain dysfunction that manifests as an altered mental state, presenting as impairment in attention, cognition, orientation, and consciousness. A retrospective study of 31 SARS-CoV-2 encephalopathy patients reported the mean age of patients as 64.6 ± 12.1 years. 60% of patients with severe encephalopathy presented with headaches, and 53.8% presented with corticospinal tract signs on neurological examination. Most of the cohort (28 of 31 patients) was intubated due to acute respiratory distress syndrome, suggesting hypoxemia-induced encephalopathy [7]. A systematic review of 24 studies involving 33 patients with SARS-CoV-2 encephalopathy showed that the common symptoms in these patients were disorientation or confusion (72.72%), decreased consciousness (54.54%), and seizures (27.27%). Neuroimaging in these patients showed no abnormalities in 51.51% of cases, and hyperintensity in white matter was found in 24.24% of cases. This is consistent with our case and other reports of cortical and subcortical white matter T2/FLAIR signal changes in SARS-CoV-2 encephalopathy patients. EEG evaluations are mostly indeterminate in these patients; however, it is sometimes necessary to exclude subclinical seizures if there is suspicion [8].

Some proposed mechanisms include direct viral infection of the CNS, neuroinflammation secondary to immune-mediated cytokine storm, injury to the brain secondary to hypoxia from lung damage, or a consequence of the higher prevalence of cardiovascular comorbidities. The dissemination of SARS-CoV-2 in the systemic circulation or across the cribriform plate can lead to cerebral involvement, as reported in some cases of SARS-CoV-2-affected patients. Within the cerebral microcirculation, sluggish movement of blood may facilitate the interaction of the SARS-CoV-2 virus spike protein with ACE2 expressed in the capillary endothelium. Damage to the endothelium lining may provide a port of entry for the virus into the brain parenchyma. Once within the neuronal tissues, its interaction with the ACE2 receptor expressed in neurons can initiate a cycle of viral budding and neuronal damage without substantial inflammation, as evidenced by some cases of SARS-CoV-2 [9]. In animal models, the coronaviruses have been shown to proliferate in the limbic structure, which supports the association of COVID-19 with psychiatric manifestations [10].

Remdesivir, formerly GS-5734, is a broad-spectrum antiviral that the Food and Drug Administration approved for the management of hospitalized SARS-CoV-2-positive patients. It is a prodrug of an adenosine analog. It causes chain termination by competing with endogenous ATP at the time of incorporation into viral RNA by RNA-dependent RNA polymerase. After administration, it is taken up by the cell and converted to an alanine metabolite, followed by conversion to nucleoside monophosphate (NMP) and subsequently to nucleoside triphosphate (NTP) through phosphorylation. The newly formed NTP is then incorporated into RdRp, causing inhibition of viral RNA replication. Due to complete first-pass metabolism through the liver, remdesivir is not suitable for oral administration and is thus administered intravenously [11]. An international double-blind, randomized, placebo-controlled trial, known as the Adaptive Covid-19 Treatment Trial (ACTT-1), included 1062 SARS-CoV-2 positive patients, with 541 patients allocated to the remdesivir arm and 521 patients allocated to the placebo arm. Remdesivir was administered intravenously as a 200-mg loading dose on day one, followed by a 100-mg daily maintenance dose on days two through 10. Remdesivir was superior to placebo in shortening the time to recovery, and its benefit was reported to be larger when given earlier in the illness. Remdesivir was associated with a reduced mortality rate and a significant reduction in the median hospital length of stay (12 days vs. 17 days). The most common adverse events reported in the remdesivir group vs. placebo were anemia (16.5% vs. 21.7%), a decline in renal function (16.0% vs. 20.3%), hyperglycemia (13.7% vs. 11.8%), and increased liver aminotransferases (6.0% vs. 10.7%) [12].

A combination of dexamethasone and remdesivir is only indicated in those patients who require an increasing amount of supplemental oxygen or require oxygen delivery through non-invasive ventilation. According to NIH, the panel recommends against the use of dexamethasone with a class AIIa evidence (A: Strong recommendation; IIa: Moderate quality of evidence-randomized trials and sub-group analysis of randomized trials that do not meet the criteria of I rating) with an AIII (A: Strong recommendation; III: Expert opinion) for systemic corticosteroids used in hospitalized patients who do not require oxygen [13]. In addition, some adverse effects of steroids can worsen the course of encephalopathies, such as psychosis, hyperglycemia, psychomotor restlessness, delirium, anxiety, agitation, and euphoria. Furthermore, according to the RECOVERY trial, dexamethasone use among hospitalized patients with COVID-19 who did not require supplemental oxygen showed no effect on mortality (rate ratio 1.19; 95% CI, 0.91-1.5) [14]. Data from Medicare and Food and Drug Administration (FDA) revealed a marked increase prescription of systemic corticosteroids among non-hospitalized patients with COVID-19 despite a lack of safety and efficacy data on the use of systemic corticosteroids. On the contrary, dexamethasone use is associated with increased mortality in COVID-19-positive hospitalized patients who do not require oxygen as per the observational cohort of US veterans [13-14].

## Conclusions

COVID-19 encephalopathy is frequently encountered in hospital settings. Due to the absence of an obvious indication and the undesirable effects associated with steroid use, we proceeded with treating the patient with remdesivir without dexamethasone. The authorities should ensure that the latest resources are available to the providers to prevent overtreating the patients. Additionally, we should treat our patients by practicing nonmaleficence, i.e., "Do no harm," as one of the core ethical principles in patient care.
